# Ultrasound as a Screening Tool for Performing Caudal Epidural Injections

**DOI:** 10.5812/iranjradiol.13262

**Published:** 2014-05-15

**Authors:** Mahshid Nikooseresht, Masoud Hashemi, Seyed Amir Mohajerani, Farideh Shahandeh, Mahvash Agah

**Affiliations:** 1 Department of Anesthesiology, Hamadan University of Medical Sciences, Hamedan, Iran; 2 Department of Anesthesiology, Shahid Beheshti University of Medical Sciences, Tehran, Iran; 3 Department of Radiology, Akhtar Hospital, Shahid Beheshti University of Medical Sciences, Tehran, Iran

**Keywords:** Anesthesia, Caudal, Ultrasonography, Low Back Pain

## Abstract

**Background:**

The caudal approach to the epidural space has been used for decades to treat low back pain caused by lumbosacral root compression. The use of fluoroscopy during epidural steroid injection is the preferred method for placing the needle more accurately in the sacral hiatus, but it carries the risk of radiation hazard.

**Objectives:**

The aim of the study was to assess the anatomical structure of the sacral hiatus and the feasibility of caudal epidural injections under ultrasound guidance.

**Patients and Methods:**

Two hundred and forty patients (male = 100, female = 140) with low back pain and sciatica who were candidates for caudal epidural injection were enrolled into this study. Ultrasound images of the sacral hiatus and bilateral cornua were obtained by a real-time linear array ultrasound transducer. The distance between bilateral cornua and the anterior and posterior wall of the sacrum were measured at the base (sacral hiatus). Under the guide of ultrasonography, we defined the injection successful if turbulence of medication fluid was observed in the sacral canal, but correct placement of the needle and injectant was confirmed on fluoroscopic view as the gold standard technique.

**Results:**

The epidurogram showed that the injection was successful in 230 of the 240 patients (95.8%). In eight patients, the injection was not in the correct place in the sacral canal. The sacral hiatus could not be identified by ultrasound images in only two patients who had a closed sacral hiatus identified by fluoroscopy. The mean distance of the sacral hiatus was 4.7 ± 1.7 mm and the mean distance between bilateral cornua was 18.0 ± 2.8 mm. The mean duration of the procedure was 10.8 ± 6.8 minutes. No major complication was observed in the next month.

**Conclusions:**

In conclusion, ultrasound could be used as a safe, fast and reliable modality to observe the anatomic variation of the sacral hiatus and to perform caudal epidural injections.

## 1. Background

Ultrasound-guided injections may have advantages over traditional techniques for the performance of regional anesthesia. Practitioners have reported less vascular puncture, more frequent success, and a reduced dose of local anesthetic with the use of ultrasound (1, 2). Epidural injection of corticosteroids has been used as an accepted nonsurgical treatment in managing chronic low back pain (3). Fluoroscopic transforaminal injection of steroids into the epidural space remains the most accurate and effective route of drug administration, in which medication was administered in the ventral part of the epidural space near the spinal nerve root (4, 5). Caudal epidural injection is an easy and safe way to administer drug in the outpatient setting with a lower risk of thecal sac puncture (6). In the caudal approach, the epidural space is entered via the sacral hiatus, so anatomical variations of the sacrum and abnormalities of the sacral hiatus arechallenges during caudal injections making it difficult to locate the sacral hiatus (7) in adults that instigate clinicians to use fluoroscopy as the gold standard method for confirming the correct needle position (8). The major limitation to caudal approach is the high missing rate of blind injections. The literature suggests rates of incorrect needle placement could be between 14-56% in non-radiologically guided caudal epidural injections (9). Fluoroscopy confirms that the needle is in the correct place and that medications are properly injected into the epidural space, but it poses radiation hazard to the patient and the interventionist and it may not be feasible in everyday practice (10, 11).

In recent years, ultrasound has been used widely for regional blocks and assessment of the anatomy of the nervous system. Application of ultrasound as a safe and fast modality to locate the sacral hiatus and to guide needle placement in caudal epidural injection has been reported (12-15). Ultrasound can provide clear images of the sacral hiatus and detect the anatomic variations of the sacrum and sacral hiatus that makecaudal epidural injection difficult or impossible.

## 2. Objectives

We performed this study for two purposes: first to investigate whether ultrasound can be used as a screening tool to predict the missed rate of caudal epidural injections and second to examine the feasibility of employing real-time US to guide the needle into the caudal epidural space.

## 3. Patients and Methods

### 3.1. Patient Selection

The study was reviewed and approved by the ethics committee of Shahid Beheshti University of Medical Sciences and it had been performed in accordance with the ethical standards laid down in an appropriate version of the 2000 declaration of Helsinki. Information about trial was given comprehensively both orally and in written form to all patients or their accompanying adult. They gave their informed consents prior to their inclusion in the study according to University Hospital Ethical Board Committee. The patients were fully informed of the risks and expected benefits of caudal epidural injections and were monitored for one month after the procedure for unexpected complications. Two-hundred fifty patients with low back pain and radicular pain in the lower extremity aged between 35 and 90 years from September 2010 to August 2012 in the pain clinic of Akhtar hospital were enrolled into the study. Ten patients refused to participate, so 240 patients (100 males and 140 females) were included. Patients were enrolled in the study if they had low back pain and radicular pain (Visual Analogue Scale ≥ 3) of more than one month duration with no response to conservative management. Exclusion criteria were as follows: symptoms requiring emergency surgery, any active sensory or motor deficit (mild chronic motor deficit or mild paresthesia were not excluded), peripheral nerve lesions, the history of recent trauma and fracture, long standing medical conditions including diabetes and cardiac disease, infection at injection site, coagulopathy, allergy to iodinated contrast or medications and pregnancy.

### 3.2. Ultrasound and Caudal Anesthesia

We collected the following data for each patient: age, gender, weight, height and symptom duration. The Honda electronics, HS 2600 ultrasound machine with 10 MHz linear-array ultrasound transducer was used in this study. The patients were placed in the prone position. The bony landmark of the sacral hiatus, located between bilateral cornua, was palpated by hand and marked subsequently. Then, using ultrasound, two sacral cornua were identified as two hyperechoic reverse U shaped structures and the distance between the apex of one sacral cornua to the other one was measured on the transverse view. On longitudinal view, the distance between the anterior wall and posterior wall of the sacral hiatus was measured in the sacral hiatus apex area.

The pain specialist set up the equipment needed for injection on a table and the ultrasound probe was covered with sterile plastic. A wide area of the back skin from the iliac crest margin to the lower buttock was cleansed three times using povidone-iodine, and then covered with a sterile drape. The skin of the injection site and subcutaneous tissue were infiltrated with 1% lidocaine. A 22 gauge spinal needle was placed in line with and parallel to the transducer (ultrasound beam). The needle shaft was visualized and under the guide of ultrasound was advanced into the sacral hiatus using the longitudinal section. Passage of the needle through the sacral hiatus was observed by the operator. When the operator was satisfied that the needle was in the sacral hiatus, 5 ml of iodinated contrast agent was injected and the turbulence of the injected material was observed in the sacral canal under the guide of ultrasound. Then an anteroposterior fluoroscopic view was obtained to check that the contrast agent filled the epidural space and filling of the epidural space on the fluoroscopic view was defined as procedural success. Once proper needle placement was established, 80 mg triamcinolone mixed in a total volume of 15 mL bupivacane 0.125% was injected. Patients were monitored for one hour in the pain clinic and asked about the immediate adverse events such as vasovagal reaction, facial flushing or severe pain during injection. Adverse reactions were assessed during the one week and one month follow-up visits.

### 3.3. Statistical Analysis

All multiple comparison tests were two-tailed. Direct comparisons between the two treatment groups were performed with the unpaired Student t-test or the nonparametric Mann-Whitney test when the data sets were not normally distributed. P value of 0.05 or less was considered significant. All statistical analyses were performed with SPSS for Windows version 18 (SPSS Inc., Chicago, Ill, USA).

## 4. Results

We included 240 patients (100 males and 140 females) with a mean age of 65.4 ± 14.6 years (range, 36-90). The mean duration of the procedure was 10.8 ± 6.8 minutes (range, 5-30). The clinical diagnosis was spinal canal stenosis in 141 patients (58.8%), lumbar disc herniation in 50 patients (20.8%), degenerative joint disease in 15 (6.2%), and failed back surgery in 30 patients (12.5%). Four patients (1.7%) had other diagnoses. The mean body mass index (BMI) was 27.2 ± 4.8 kg/m^2^ (range = 17-37). According to the BMI categorization for Asian populations by WHO guidelines, nearly 60% of our patients were overweight or preobese (BMI > 25). The age and other characteristics of the patients are listed in Table 1. BMI, sex or age of the patients was not significantly related to the success of US-guided caudal injection (All Ps > 0.05).

**Table 1.** Patient’s Characteristics

	Mean±SD	Min-Max
**Age, y**	65.4±14.6	36-88
**Body Mass Index, kg/m^2^**	27.18±4.8	17-37
**Distance Between Bilateral Cornea, mm**	18±2.8	10.4-22.1
**Distance of Sacral Hiatus, mm**	4.7±1.7	1.5-8.9

**Figure 1.**
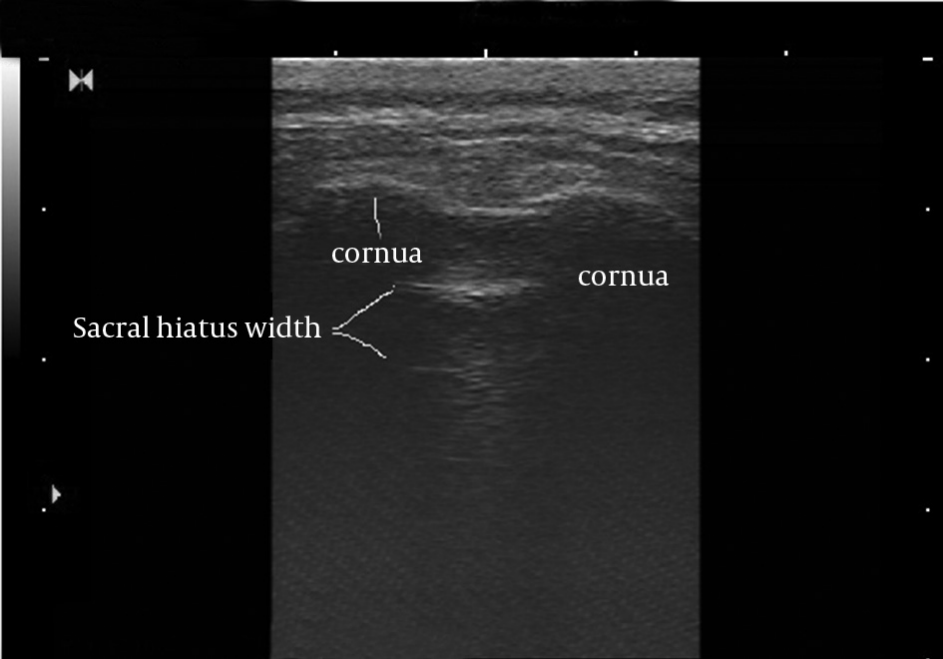
Transverse view of the sacral hiatus by ultrasound; the bilateral cornua are seen as reverse U-shaped structures.

**Figure 2.**
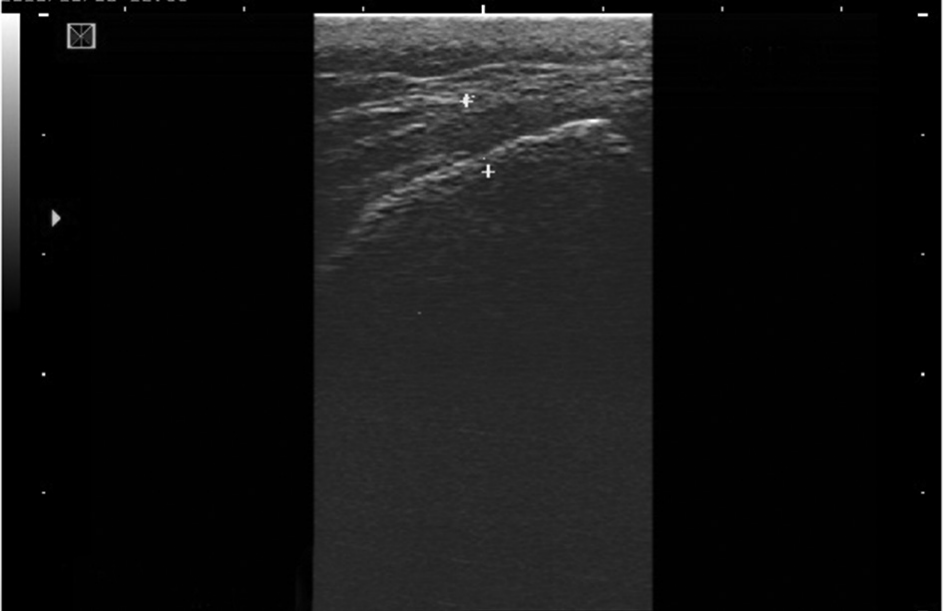
Longitudinal view of the sacral hiatus by ultrasound, two points refer to the diameter of the sacral canal at the apex.

Bony landmarks of the sacral hiatus could be palpated in 205 patients (85.4%). The sacral hiatus was identified in 238 of 240 patients (99.1%) by ultrasound images. In two patients (0.8%), visualization of the sacral hiatus was not possible by ultrasound and a closed sacral hiatus was identified on the lateral sacral bone x-ray. The mean diameter of the sacral canal at the apex of the sacral hiatus (sacral hiatus distance) was 4.7 ± 1.7 mm (range, 1.5-8.9). The mean distance between the apexes of the two sacral cornua was 18 ± 2.8 mm (range, 10.4-22.1) (Figures 1 and 2).

**Figure 3.**
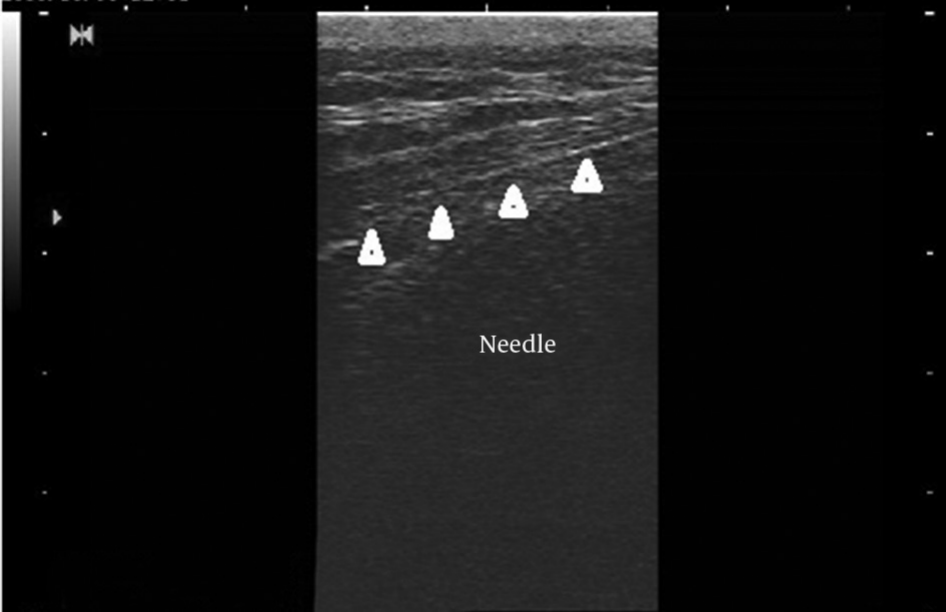
Needle in the sacral canal on the longitudinal view; arrowheads refer to the needle.

**Figure 4.**
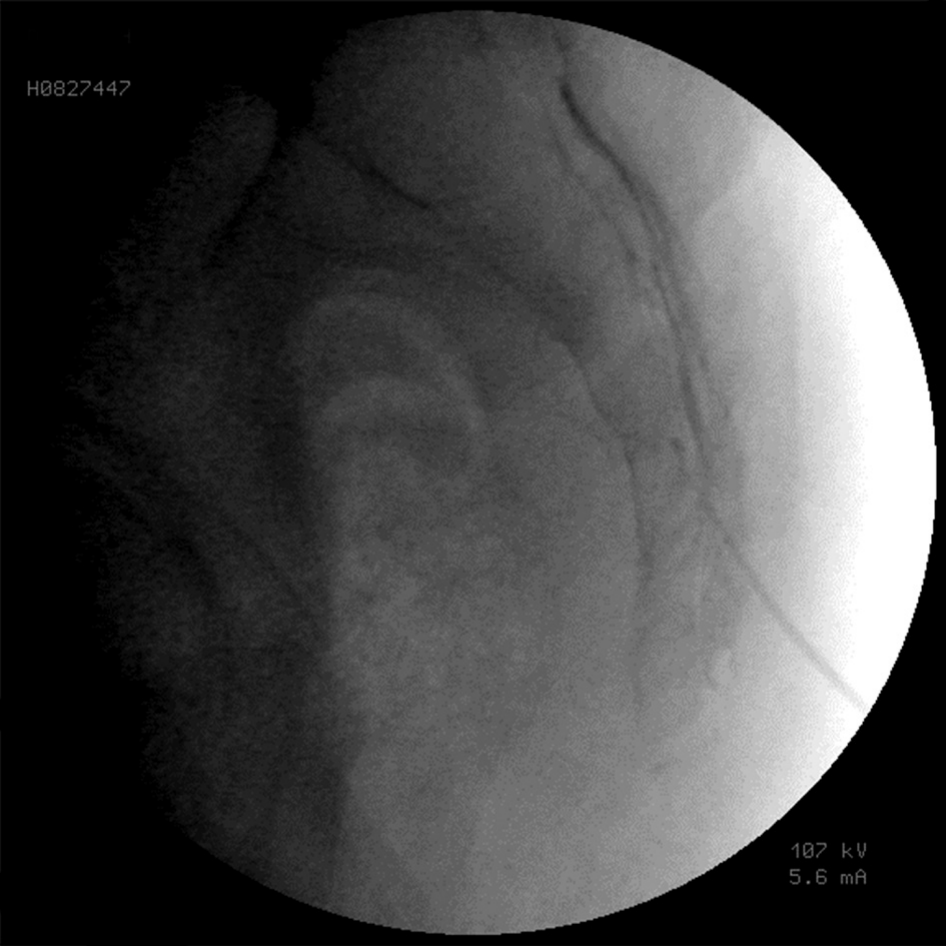
Fluoroscopic view of the contrast injected in the sacral canal

**Figure 5.**
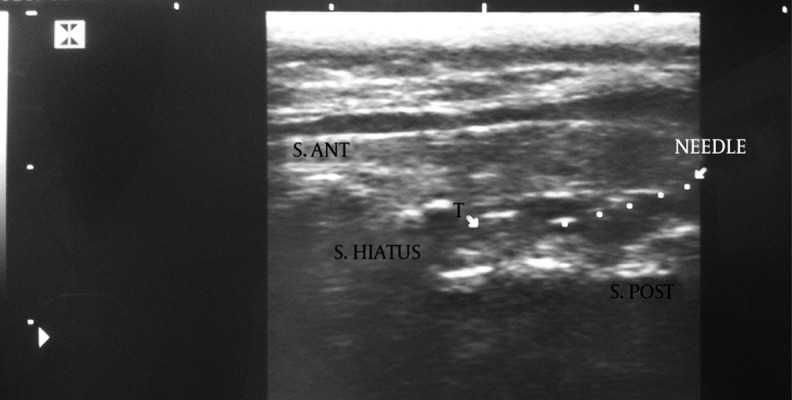
Turbulence of the injected fluid in the sacral canal; T=Turbulence, the dashed line indicates the needle in the sacral hiatus.

The epidurogram showed that the injection under ultrasound guidance was successful in 230 of 240 patients (95.8%). Comparing means with independent sample t-test, the mean diameter of the sacral hiatus in patients with failed contrast injection was 1.61 ± 0.1 mm that was significantly lower than the mean diameter of the hiatus in patients with successful US guided injection (4.7 ± 1.7mm) (P< 0.001) (Figures 3, 4 and 5).

The mean bicornual distance in successful and failed US-guided injections were 17.9 ± 2.7 mm and 19.7 ± 1.5 mm, respectively (P = 0.1). The distances between bilateral cornua were 19.6 mm and 21.7 mm in two patients with failed caudal epidural injection in our study in whom we were not able to see the sacral hiatus by ultrasound. There were no major complications related to the regional anesthetic technique in the next month. No severe pain, muscle weakness, hemodynamic instability or any other adverse event was observed. Twenty-eight patients (11.6%) had minor complications such as mild and moderate pain during needle insertion and advancement in 20 patients (8.4%), nausea in three patients (1.2%), flushing in 14 patients (5.9%) and mild lightheadedness, weakness and fatigue were seen in three patients (1.2%) that lasted up to one week (Figures 1-5). 

## 5. Discussion

Sacral hiatus is the most important bony landmark for performing caudal epidural injections and the success rate of the caudal epidural injection depends on the correct placement of the needle in the sacral canal and anatomic variations of the sacral hiatus (12). The anatomy of the sacral hiatus has been studied in several studies and based on radiologic or cadaveric measurements, some points were suggested to increase the safety and success rate of caudal injections (16). The mean distance between the hiatal apex and the dural sac has been reported to be 45-60.5 mm and the mean sacral space depth has been observed to be 4.6 mm in adults. Lower insertion angles have been suggested in infants with respect to adult subjects (21˚ vs. 58˚) (17). It has been mentioned in another cadaveric study that dimensions of the triangle formed by the right and left posterosuperior iliac spines with the apex of the hiatus, the optimal angle of needle insertion and the depth of the caudal space are mainstays of anatomical landmarks (18). Morphometric characteristics of the sacral hiatus in 46 Egyptian dry sacra clarifies that a less than 3 mm anteroposterior diameter of hiatus in Egyptian females and an absent sacral hiatus in Egyptian males should be taken into consideration before caudal epidural block to avoid its failure (19).

Anatomical details of the sacral hiatus, bilateral sacral cornua, apex of the sacral hiatus, anterior and posterior walls of the sacral canal and sacrococcygeal ligament can be clearly detected under the guide of ultrasound (9, 12, 20). Our study provides detailed knowledge of the anatomy of the sacral hiatus and practical landmarks in a large number of Iranian patients (n = 240). We were not able to palpate the bony landmark of the bilateral cornua in 35 patients (14.5%) consistent with the findings of Chen (14.3%) and colleagues (12). Using ultrasound, sacral hiatus details could be seen in 238 of 240 patients and this means that ultrasound is a valuable tool for the assessment of bony landmarks in caudal injection. The diameter of the sacral canal at the apex of the sacral hiatus was measured to have a mean of 4.7 ± 1.7 mm in our study versus 9.7 ± 1.9 mm and 14.2 ± 3.5 mm in Chen and Blanchais studies, respectively (9, 12). These different results may be attributed to racial diversity, size measurement discrepancy due to the ultrasound technique or physician expertise. In two of our 10 patients with failed caudal epidural injections, the sacral hiatus could not be identified by ultrasound images, while using a fluoroscope was also unsuccessful. Lateral x-ray of the sacrum in these patients showed that the sacral hiatus was anatomically closed. Therefore, closed sacral hiatus frequency was 0.8% of our patients in comparison to the findings of Sekiguchi and colleagues, who studied 92 cases and found a closed hiatus in 3% and an absent hiatus in 4% of their cases (21). In the study performed by Chen et al. ultrasound images revealed a closed sacral hiatus in one of the 47 patients (2%) (12). It has been reported that 5-10% of the patients have abnormality of the sacral hiatus that makes cannulation difficult or impossible (11).

In eight patients with failed injection, the sacral canal diameter was measured to be between 1.5 and 1.9 mm. It was difficult to insert the needle into the sacral canal and severe pain and soreness was felt during the procedure in five patients. Epidurogram showed that the injection was out of the sacral canal in these eight patients. It has been reported that sacral canal diameters of 2 mm or less (21), around 1.5 mm (12) or less than 3 mm (19) can result in the increased failure rate of caudal epidural injections and a sacral canal diameter of 2.9 mm is adequate for performing successful injection (12). These findings were in agreement with the results of our study that a sacral hiatus diameter of 1.5-1.9 mm results in failed caudal epidural injection. Based on the results of our study and similar studies (9, 12), the distance between bilateral cornua is not a predicting factor for causing failed caudal epidural injection.

BMI, gender or the age of the patients was not significantly related to the success of caudal injection. Apparently, anatomical variations are not related to BMI, age or the gender of the patients and innately are not predictable by the patients’ demographic characteristics. Based on the BMI categorization for Asian populations by WHO guidelines, 67.6% of our patients were mildly overweight or preobese, but we did not observe excessive fat tissue overlying the sacrum to make the anatomic details of the sacral hiatus invisible. Similar results have been reported by Chen, in which clear ultrasound images of the sacral hiatus could be obtained in patients with a BMI range of 23-27 kg/m^2^ (12).

The high success rate (95.8%) of caudal epidural injection under ultrasound guide was conclusive in our study, which was in agreement with the findings of Yoon et al. that the procedural success rate was reported as high as 94% (11). Successful ultrasound-guided injection in caudal epidural space was reported in 85.1% and 90% of the patients in two other studies (9, 12). The high success rate in our study and similar studies reinstated that ultrasound could be an effective device appropriate for caudal injection without the risk of radiation hazard for the patient and the pain specialist. In our study, the obtained ultrasound images suggested that ultrasound may be used as an effective tool in assessing the anatomic variations of the sacral hiatus and judging whether caudal epidural injection can be performed successfully on a patient. Observation of needle advancement in the sacral canal, turbulence of the injected fluid and negative bloody aspiration can be used as indicators of successful injection, but ultrasound cannot provide the image information about the exact depth of the inserted needle and there is no way to totally rule out the intravascular injection under ultrasound guidance.

In conclusion, ultrasound is an effective tool, not only to guide the insertion of the needle into the caudal epidural space, but also to predict the procedural success rate. Ultrasound-guided caudal block significantly improves the success rate in patients and besides it decreases the time consumed for fluoroscopy-guided injections. Closed sacral hiatus or sacral canal diameters of less than 2 mm on ultrasound images may suggest a high incidence of unsuccessful caudal epidural injection.
